# The Dynamics of Lateral Gene Transfer in Genus *Leishmania* - A Route for Adaptation and Species Diversification

**DOI:** 10.1371/journal.pntd.0004326

**Published:** 2016-01-05

**Authors:** Elisabet Vikeved, Anders Backlund, Cecilia Alsmark

**Affiliations:** 1 Division of Pharmacognosy, Department of Medicinal Chemistry, Biomedical Centre, Uppsala University, Uppsala, Sweden; 2 Department of Microbiology, National Veterinary Institute (SVA), Uppsala, Sweden; Seattle Biomedical Research Institute, UNITED STATES

## Abstract

**Background:**

The genome of *Leishmania major* harbours a comparably high proportion of genes of prokaryote origin, acquired by lateral gene transfer (LGT). Some of these are present in closely related trypanosomatids, while some are detected in *Leishmania* only. We have evaluated the impact and destiny of LGT in genus *Leishmania*.

**Methodology/Principal Findings:**

To study the dynamics and fate of LGTs we have performed phylogenetic, as well as nucleotide and amino acid composition analyses within orthologous groups of LGTs detected in *Leishmania*. A set of universal trypanosomatid LGTs was added as a reference group. Both groups of LGTs have, to some extent, ameliorated to resemble the recipient genomes. However, while virtually all of the universal trypanosomatid LGTs are distributed and conserved in the entire genus *Leishmania*, the LGTs uniquely present in genus *Leishmania* are more prone to gene loss and display faster rates of evolution. Furthermore, a PCR based approach has been employed to ascertain the presence of a set of twenty LGTs uniquely present in genus *Leishmania*, and three universal trypanosomatid LGTs, in ten additional strains of *Leishmania*. Evolutionary rates and predicted expression levels of these LGTs have also been estimated. Ten of the twenty LGTs are distributed and conserved in all species investigated, while the remainder have been subjected to modifications, or undergone pseudogenization, degradation or loss in one or more species.

**Conclusions/Significance:**

LGTs unique to the genus *Leishmania* have been acquired after the divergence of *Leishmania* from the other trypanosomatids, and are evolving faster than their recipient genomes. This implies that LGT in genus *Leishmania* is a continuous and dynamic process contributing to species differentiation and speciation. This study also highlights the importance of carefully evaluating these dynamic genes, e.g. as LGTs have been suggested as potential drug targets.

## Author Summary

*Leishmania* parasites cause leishmaniasis, a neglected tropical disease, estimated to threaten 350 million people in 88 countries worldwide according to the WHO. The genome of *Leishmania major* harbours a number of genes, which have been proposed as acquired by lateral gene transfer (LGT) from a broad variety of prokaryote donors. Such genes may prove beneficial for the parasites, e.g. by promoting survival of the parasite in new environments. We have studied orthologs to LGTs previously detected uniquely in *L*. *major* as well as LGTs shared also by other trypanosomatids. The universal trypanosomatid LGTs are more conserved within genus *Leishmania*, as compared to LGTs that are exclusively present in genus *Leishmania*. One possible explanation to this observation is that these have resided in their host genomes for a longer time period. This explanation strengthens the hypothesis that the LGTs unique to genus *Leishmaina* were acquired after the divergence from the trypanosomes, rather than before the divergence, and then subsequently lost from the trypanosome lineage. An in-depth analysis of a subset of the LGTs, which are present only in genus *Leishmania* showed that LGT within genus *Leishmania* is a dynamic process. LGTs, providing beneficial capabilities to the parasite, are demonstrated to become conserved throughout generic diversification, hence contributing to species differentiation, while LGTs of limited use are degraded and lost.

## Introduction

Trypanosomatids are single flagellated, kinetoplastid protozoa with parasitic lifestyles. Primary hosts are often insects [1], but secondary hosts may be vertebrates and some species, such as the sister genera *Leishmania* and *Trypanosoma*, cause major diseases in humans [2]. The kinetoplastids uniquely harbour the kinetoplast, a tubular network of interlocked circular mitochondrial DNA. Based on several molecular markers [3–7], genus *Leishmania* is divided into the three subgenera *Leishmania*, *Viannia* and *Sauroleishmania*. Fraga and co-workers (2010) further sub-divides these three subgenera into several informal complexes, in general congruence with most previous studies. The main advancements in the most recent classifications is the separation of the *major*- and *tropica*-complexes, and the erection of a *guyanensis*-complex encompassing strains previously included in the *braziliensis*-complex [3, 4].

Species of genus *Leishmania* causes leishmaniasis, a neglected and largely tropical disease, threatening the health of 350 million people in 88 countries worldwide according to WHO estimates [8]. The zoonotic parasite is transferred to humans and animals via the bite of several species of sand flies of subfamily *Phlebotominae* [9]. From the complete sequencing of the genome of *Leishmania major* [10] it became evident that this genome includes a number of genes acquired by lateral gene transfer (LGT) from prokaryote donors [11–13]. LGT implies the uptake and fixation in a genome of DNA originating from another, distantly related, organism. LGT has been recognized to contribute to genome evolution in prokaryotes [14, 15] and eukaryotes alike [16–18], and offers a rapid route to the acquisition of new capacities, such as utilization of alternative metabolites or adaptation to new environmental niches [19, 20]. The mechanism(s) of LGT in eukaryotes is not known, although phagosytosis by means of protozoan grazing [21], uptake via transposable elements [22–24] and donations from symbionts [25] have all been suggested. The broad collection of donor organisms within our dataset [11, 12] speaks against one, single event of acquisition from one single symbiotic donor. The majority of donors inferred by phylogeny are proteobacteria, and these are abundant in the gut of the insect host of *Leishmania*. However, since we predict that most transfer events preceded the radiation of genus *Leishmania*, assumptions on putative hosts of this ancestral *Leishmania* are uncertain. LGT between prokaryotes have been shown to be transient, and although some LGTs are fixed in the host genomes and passed on after divergences into new species, these fixed LGTs are outnumbered by genes rapidly gained and lost [18, 26, 27]. Whether or not this is also the case for LGTs in eukaryote genomes, has not been completely elucidated [18], but recent studies have shown that eukaryote genomes can acquire long stretches of prokaryotic genes, from which beneficial genes are conserved and superfluous genes rapidly discarded [24]. With the aim to determine the dynamics of LGT generally and the depth of the transfer within genus *Leishmania* specifically, we have analysed orthologous groups of LGTs, previously confirmed with phylogenomic analyses in *L*. *major* only [28–30], and also in the genomes of the close relatives *Trypanosoma brucei* and *Trypanosoma cruzi* [11, 12]. Furthermore, we have used a PCR-based approach to investigate ten additional strains of *Leishmania* parasites with unpublished genomes, for the presence of a subset of twenty LGTs that are unique to genus *Leishmania*. For these genes we have evaluated features of amino acid and nucleotide composition, as well as phylogeny in order to gain increased understanding of the fate of transferred prokaryotic genes in their new eukaryotic genomic environments. LGTs have been suggested as potential drug targets in trypanosomatids [12] and careful evaluation of the stability of potential drug targets is an essential initial step towards the proposal of future drugs to treat infection.

## Materials and Methods

### Data mining and sequence retrieval

Orthologous sequences of the LGTs previously confirmed with phylogenomic analyses in *L*. *major* [11, 12] were retrieved from the completely sequenced genomes of *L*. *braziliensis L*. *infantum*, *L*. *mexicana*, *L*. *tarentolae*, *T*. *brucei* and *T*. *cruzi* using homology searches with the BLAST [31] tool in TriTrypDB v 6.0 [32].

### Sequence similarity and G+C content

The average amino acid and nucleotide identities were calculated between each LGT and orthologs in *L*. *braziliensis*, *L*. *infantum* and *L*. *tarentolae* respectively. Identity scores of LGTs were compared to the calculated average amino acid and nucleotide identity between the coding content of each complete genome [28, 29].

The G+C content was calculated for each LGT and orthologs in *L*. *braziliensis*, *L*. *infantum* and *L*. *tarentolae* respectively. The G+C content of LGTs was compared to the coding G+C content of each complete genome [28, 29].

Significance differences were calculated using Wilcoxon Signed-Rank Test (p<0,05).

### DNA preparation

Frozen cell cultures of *Leishmania* promastigotes from ten different strains of *Leishmania* were kindly provided by the Public Health Agency of Sweden (Folkhälsomyndigheten) as listed in Table 1. Genomic DNA was extracted from the *Leishmania* promastigote cell cultures using Qiagen Blood & Tissue KIT (Qiagen, Sollentuna, S) according to the manufacturer’s instructions. The protocol for “Purification of Total DNA from animal Blood or Cells (Spin-Column Protocol)” was used from step 1c “Cultured cells” and onwards.

**Table 1:** Strains of *Leishmania* included in this study.

Subgenus	Complex	Strain	Strain abbreviation
***Viannia***	*braziliensis*	*Leishmania braziliensis* LB2904 MHOM/BR/75M2904^a^	LbrM
***Viannia***	*braziliensis*	*Leishmania braziliensis* L2346/05	LbrL
***Viannia***	*braziliensis*	*Leishmania braziliensis* complex L2237/05^e^	LbrcXL
***Viannia***	*guyanensis*	*Leishmania panamensis* MHOM/PA/71/LS94	LpnM
***Viannia***	*guyanensis*	*Leishmania panamensis* L967/96	LpnL
***Sauroleishmania***	*tarentolae*	*Leishmania tarentolae* Parrot-TarII ^b^	LtaP
***Leishmania***	*mexicana*	*Leishmania mexicana* MHOM/GT/2001/U1103 ^c^	LmxM
***Leishmania***	*mexicana*	*Leishmania mexicana* MHOM/BZ/82/BEL21	LmxMB
***Leishmania***	*mexicana*	*Leishmania amazonensis* HSJD-1	LamH
***Leishmania***	*major*	*Leishmania major* Friedlin ^*d*^	LmjF
***Leishmania***	*major*	*Leishmania major* L1989/05	LmjL
***Leishmania***	*tropica*	*Leishmania tropica*	LtrX
***Leishmania***	*donovani*	*Leishmania infantum* JPCM5 MCAN/ES/98/LLM-877 ^*a*^	LinJ
***Leishmania***	*donovani*	*Leishmania infantum* MHOM/00/97/3277	LinM
***Leishmania***	*donovani*	*Leishmania donovani* LV9	LdoL

^a^ [28]

^b^ [29]

^c^ [30]

^d^ [10]

^e^ It is verified that the promastigote belongs to the *braziliensis* complex, however it is not stated what species it belongs to.

DNA concentrations were measured with a Thermo Scientific NanoDrop 2000c Spectrophotometer (Thermo Fisher Scientific, Wilmington, DE), according to the manufacturer’s instructions for pedestal measurement.

### Primer design

Oligonucleotide primers for amplification of orthologs to the LGTs were primarily designed based on the sequences and flanking regions of LGTs in the published genome of *L*. *major*. In some cases, oligonucleotide primers where also designed for the orthologs of the LGTs in the other *Leishmania* strains for which there are published genomes. Where needed multiple primer pairs were designed to amplify LGTs. The primers were designed in Geneious v.5/v.6 (Biomatters, Auckland, NZ) using the Primer3 algorithm [33, 34]. Detailed information about the oligonucleotide primers can be found in S1 Table.

### PCR

Polymerase chain reactions were performed in a total reaction volume of 50 μl, containing Taq DNA Polymerase Master Mix (75 mM Tris-HCl pH 8.5, 20 mM (NH_4_)_2_ SO_4_, 2.0 mM MgCl_2_, 0.2 mM dNTPs, 0.1% Tween 20 and 1.25 U Ampliqon *Taq* polymerase (VWR International, Radnor, PE), 0.2 μM of each primer (Sigma Aldrich, St. Louis, MO), 5–20 ng genomic DNA and double distilled, sterile filtered milliQ water (Merck Millipore, Billerica, MA). The DNA was amplified using an Eppendorf Mastercycler personal (Eppendorf, Hamburg, D) and a Bio-Rad C1000TM thermal cycler (Bio-Rad, Hercules, CA) under the following conditions: initial denaturation at 95°C for three minutes, followed by 35 cycles with denaturation at 95°C for 30 seconds, annealing at the annealing temperatures, Ta, optimized for the different oligonucleotide primer pairs (S1 Table) for 60–90 seconds, elongation at 72°C for 60 seconds and a final extension step at 72°C for 10 minutes. Positive amplifications were confirmed on a MCE-202 MultiNA instrument, a microchip electrophoresis system for DNA and RNA analysis (Shimadzu Biotech, Kyoto, J), in accordance with the manufacturer’s instructions.

### Sequencing

The amplicons were purified with the QIAquick PCR purification Kit (250), (Qiagen, Düsseldorf, D), according to the manufacturers instruction. Purified amplicons were subsequently sequenced by Macrogen on an ABI3730xl platform (Macrogen, Seoul, KR).

### Phylogenetic analysis

The sequencing results were assembled using Geneious v.5/v.6 (Biomatters, Auckland, NZ) and the obtained sequences were named with the strain abbreviation (Table 1) followed by the chromosome and gene location of the LGTs in *L*. *major F*.

All obtained sequences were uploaded to GenBank and allocated the following accession numbers; KM411704-KM411876. The sequences where aligned using MUSCLE [35] and the alignments were manually inspected and refined.

All phylogenetic analyses were performed using the software PAUP* 4.0b10 for UNIX [36, 37] on an Apple MacBook Pro. Under a Fitch parsimony optimality criterion 1000 random addition sequences were followed by “tree bisection-reconnection” branch-swapping saving all optimal trees [38]. Support analyses were performed with 10000 bootstrap replicates, each consisting of three random addition sequences and subsequent “subtree pruning and regrafting” branch-swapping [39].

### Synteny analysis–LGTs remnants identification

The flanking genes of the LGTs that lack orthologs in one or several of the already published genomes of *L*. *braziliensis*, *L*. *infantum*, *L*. *mexicana* and *L*. *tarentolae* where identified using TriTryp DB v 6.0 [32]. Any orthologs to these LGT flanking genes in *L*. *braziliensis*, *L*. *infantum*, *L*. *mexicana* and *L*. *tarentolae* where compared in terms of synteny. If the LGT flanking genes in *L*. *major* and their orthologs in above mentioned strains were syntenic, the intergenic region between the genes were aligned with the LGT and possible orthologs from other strains using MUSCLE. The alignments were manually inspected and refined in order to find remnants of LGT orthologs in strains where no orthologs are annotated.

### Rate of sequence substitution

The ratio of non-synonymous mutations (K_a_) to synonymous mutations (K_s_) were calculated for pairwise alignments of all orthologs obtained for each of the studied LGTs, using the modified Yang-Nielson method (MYN) [40] incorporated in the software package K_a_/K_s__Calculator [41]. Protein coding genes in the *Leishmania* genomes have been shown to have a strong codon bias [42–44]. MYN adopts the Tamura-Nei model, which takes codon frequency bias and differences among transitional and transversional substitution rates into consideration when calculating K_a_ and K_s_. The K_a_/K_s_ ratios between the different strains and means were compared for the different genes. The K_a_/K_s_-ratios of the LGTs were compared to the housekeeping genes heat-shock protein 70 (HSP70) and elongation factor Tu (EF-Tu) (S2 Table). The setting “standard” was chosen for the genetic code.

### Codon bias calculations

Codon adaptation index (CAI) scores were calculated using the online tool CAIcal server at http://genomes.urv.es/CAIcal [45]. Codon usage table for *L*. *major F*. was used for all orthologs, and “standard” was selected for the genetic code since the LGTs are originally prokaryote genes in a new eukaryote environment.

### Functional annotation of LGTs

The predicted functions of LGTs were retrieved from annotations of *L*. *major* genes in TriTrypDB v 6.0 [32]. All orthologues to *L*. *major* LGTs within our dataset are assumed to have the same function.

### ID numbers of genes included in this study

LmjF.01.0310, LmjF.02.0740, LmjF.03.0040, LmjF.03.0390, LmjF.03.0680, LmjF.04.0460, LmjF.06.0560, LmjF.06.1080, LmjF.06.1270, LmjF.06.1280, LmjF.07.0030, LmjF.07.0270, LmjF.09.0200, LmjF.09.0270, LmjF.10.1250, LmjF.13.0050, LmjF.13.0090, LmjF.13.0610, LmjF.14.1190, LmjF.15.0510, LmjF.15.0740, LmjF.16.0950, LmjF.17.0140, LmjF.17.1460, LmjF.18.1580, LmjF.23.0200, LmjF.23.0260, LmjF.23.0270, LmjF.23.0690, LmjF.23.0880, LmjF.23.1650, LmjF.25.1200, LmjF.25.2010, LmjF.26.0580, LmjF.26.0830, LmjF.26.2710, LmjF.27.0930, LmjF.27.2340, LmjF.27.2660, LmjF.28.0670, LmjF.28.1340, LmjF.28.1350, LmjF.28.1950, LmjF.28.2160, LmjF.28.2510, LmjF.28.2910, LmjF.29.0250, LmjF.29.0885, LmjF.29.2800, LmjF.30.0930, LmjF.30.1380, LmjF.30.2090, LmjF.30.2310, LmjF.30.2970, LmjF.30.2990, LmjF.31.0010, LmjF.31.0560, LmjF.31.0740, LmjF.31.1810, LmjF.31.2650, LmjF.31.2785, LmjF.31.2880, LmjF.31.3050, LmjF.33.0520, LmjF.33.0560, LmjF.33.0830, LmjF.33.0960, LmjF.33.0990, LmjF.33.1680, LmjF.33.1740, LmjF.33.2300, LmjF.33.2550, LmjF.33.2830, LmjF.34.0610, LmjF.34.2140, LmjF.34.2410, LmjF.34.2850, LmjF.35.1830, LmjF.35.5330, LmjF.36.0060, LmjF.36.0260, LmjF.36.0330, LmjF.36.1660, LmjF.36.2060, LmjF.36.2750, LmjF.36.4800, LmjF.36.5240, LmjF.36.5430, LmjF.36.5770, LmjF.36.5960, LinJ.01.0310, LinJ.02.0710, LinJ.03.0040, LinJ.03.0370, LinJ.03.0660, LinJ.04.0440, LinJ.06.0580, LinJ.06.1120, LinJ.06.1330, LinJ.06.1340, LinJ.07.0040, LinJ.07.0430, LinJ.09.0220, LinJ.09.0420, LinJ.10.1390, LinJ.13.0050, LinJ.13.0090, LinJ.13.0500, LinJ.14.1270, LinJ.15.0530, LinJ.15.0780, LinJ.16.0960, LinJ.17.0250, LinJ.17.1580, LinJ.18.1570, LinJ.23.0220, LinJ.23.0300, LinJ.23.0310, LinJ.23.0680, LinJ.23.0860, LinJ.23.1060, LinJ.25.1230, LinJ.25.2090, LinJ.26.0550, LinJ.26.0790, LinJ.26.2740, LinJ.27.0790, LinJ.27.2290, LinJ.28.0710, LinJ.28.1450, LinJ.28.1460, LinJ.28.2080, LinJ.28.2310, LinJ.28.2700, LinJ.28.3140, LinJ.29.0260, LinJ.29.0960, LinJ.29.2910, LinJ.30.0990, LinJ.30.1440, LinJ.30.2100, LinJ.30.2320, LinJ.30.2990, LinJ.30.3010, LinJ.31.0010, LinJ.31.0580, LinJ.31.0770, LinJ.31.1840, LinJ.31.2720, LinJ.31.2980, LinJ.31.3170, LinJ.33.0530, LinJ.33.0580, LinJ.33.0870, LinJ.33.1010, LinJ.33.1040, LinJ.33.1780, LinJ.33.1840, LinJ.33.2430, LinJ.33.2680, LinJ.33.2970, LinJ.34.0630, LinJ.34.1900, LinJ.34.2180, LinJ.34.2710, LinJ.35.1820, LinJ.35.5300, LinJ.36.0060, LinJ.36.0280, LinJ.36.0360, LinJ.36.1740, LinJ.36.2180, LinJ.36.2890, LinJ.36.5030, LinJ.36.5470, LinJ.36.5670, LinJ.36.6020, LinJ.36.6220, LmxM.01.0310, LmxM.02.0740, LmxM.03.0040, LmxM.03.0680, LmxM.04.0460, LmxM.06.0560, LmxM.06.1080, LmxM.06.1270, LmxM.06.1280, LmxM.07.0030, LmxM.07.0270, LmxM.08_29.0250, LmxM.08_29.0885, LmxM.08_29.2800, LmxM.09.0200, LmxM.09.0270, LmxM.10.1250, LmxM.13.0050, LmxM.13.0090, LmxM.13.0610, LmxM.14.1190, LmxM.15.0510, LmxM.15.0740, LmxM.16.0950, LmxM.17.0140, LmxM.17.1460, LmxM.18.1580, LmxM.23.0200, LmxM.23.0260, LmxM.23.0270, LmxM.23.0690, LmxM.23.0880, LmxM.23.1650, LmxM.25.1200, LmxM.25.2010, LmxM.26.0580, LmxM.26.0830, LmxM.26.2710, LmxM.27.0930, LmxM.27.2340, LmxM.28.0670, LmxM.28.1340, LmxM.28.1350, LmxM.28.1950, LmxM.28.2160, LmxM.28.2510, LmxM.28.2910, LmxM.29.0930, LmxM.29.1380, LmxM.29.2090, LmxM.29.2310, LmxM.29.2980, LmxM.29.2990, LmxM.30.0010, LmxM.30.0560, LmxM.30.0740, LmxM.30.2650, LmxM.30.2880, LmxM.30.3050, LmxM.32.0520, LmxM.32.0560, LmxM.32.0830, LmxM.32.0960, LmxM.32.0990, LmxM.32.1680, LmxM.32.1740, LmxM.32.2300, LmxM.32.2550, LmxM.32.2830, LmxM.33.0610, LmxM.33.2140, LmxM.33.2410, LmxM.33.2850, LmxM.34.1830, LmxM.34.5330, LmxM.36.0060, LmxM.36.0260, LmxM.36.0330, LmxM.36.1660, LmxM.36.2060, LmxM.36.4800, LmxM.36.5240, LmxM.36.5430, LmxM.36.5770, LmxM.36.5960, LbrM.01.0340, LbrM.03.0060, LbrM.03.0580, LbrM.04.0500, LbrM.06.0570, LbrM.06.1060, LbrM.06.1260, LbrM.06.1270, LbrM.07.0280, LbrM.09.0260, LbrM.10.1400, LbrM.13.0050, LbrM.13.0080, LbrM.13.0430, LbrM.14.1360, LbrM.15.0540, LbrM.15.0800, LbrM.16.0960, LbrM.17.0180, LbrM.17.1610, LbrM.18.1610, LbrM.20.0540, LbrM.20.1640, LbrM.20.1910, LbrM.23.0220, LbrM.23.0290, LbrM.23.0300, LbrM.23.0670, LbrM.23.0840, LbrM.23.0980, LbrM.25.1170, LbrM.25.1570, LbrM.26.0590, LbrM.26.0840, LbrM.26.2640, LbrM.27.1010, LbrM.27.2550, LbrM.27.2760, LbrM.28.0690, LbrM.28.1440, LbrM.28.1490, LbrM.28.2130, LbrM.28.2360, LbrM.28.2710, LbrM.28.3120, LbrM.29.0510, LbrM.29.0950, LbrM.29.2850, LbrM.30.1050, LbrM.30.2040, LbrM.30.2260, LbrM.30.2950, LbrM.30.2960, LbrM.31.0010, LbrM.31.0720, LbrM.31.0930, LbrM.31.2990, LbrM.31.3240, LbrM.31.3440, LbrM.33.0520, LbrM.33.0570, LbrM.33.1020, LbrM.33.1160, LbrM.33.1950, LbrM.33.2010, LbrM.33.2590, LbrM.33.2820, LbrM.33.3110, LbrM.34.1740, LbrM.34.5270, LbrM.35.0100, LbrM.35.0340, LbrM.35.0420, LbrM.35.1850, LbrM.35.2290, LbrM.35.2970, LbrM.35.5050, LbrM.35.5490, LbrM.35.5690, LbrM.35.6050, LbrM.35.6270, LtaP.03.0630, LtaP.06.1060, LtaP.06.1290, LtaP.10.1680, LtaP.13.0490, LtaP.15.0730, LtaP.16.0940, LtaP.23.0320, LtaP.23.0790, LtaP.26.2870, LtaP.28.0690, LtaP.30.2110, LtaP.31.3540, LtaP.33.1830, LtaP.33.1900, LtaP.34.2770, LtaP.36.0250, LtaP.36.2820, LtaP01.0310, LtaP03.0040, LtaP04.0440, LtaP06.0530, LtaP06.1280, LtaP07.0270, LtaP09.0200, LtaP09.0270, LtaP13.0050, LtaP13.0090, LtaP14.1240, LtaP15.0490, LtaP17.0220, LtaP17.1600, LtaP18.1550, LtaP23.0230, LtaP23.0330, LtaP23.0960, LtaP23.1160, LtaP25.1240, LtaP25.2130, LtaP26.0550, LtaP26.0770, LtaP27.0970, LtaP27.2460, LtaP28.1410, LtaP28.2000, LtaP28.2240, LtaP28.2590, LtaP28.2930, LtaP29.0260, LtaP29.0980, LtaP29.3010, LtaP30.1040, LtaP30.1490, LtaP30.2350, LtaP30.2970, LtaP30.2980, LtaP31.0010, LtaP31.0580, LtaP31.0770, LtaP31.3070, LtaP31.3360, LtaP33.0540, LtaP33.0590, LtaP33.0900, LtaP33.1060, LtaP33.2520, LtaP33.2780, LtaP33.3070, LtaP34.0670, LtaP34.1990, LtaP34.2260, LtaP35.1880, LtaP35.5290, LtaP36.0040, LtaP36.0320, LtaP36.1650, LtaP36.2070, LtaP36.4940, LtaP36.5360, LtaP36.5570, LtaP36.5920, LtaP36.6120, Tb927.1.3000, Tb927.10.11190, Tb927.10.11590, Tb927.10.4580, Tb927.10.4730, Tb927.10.5070, Tb927.10.6090, Tb927.10.6560, Tb927.10.6880, Tb927.10.9030, Tb927.10.9240, Tb927.10.9390, Tb927.11.10670, Tb927.11.11780, Tb927.11.12270, Tb927.11.1540, Tb927.11.2500, Tb927.11.2730, Tb927.11.4700, Tb927.11.8440, Tb927.2.3440, Tb927.3.1360, Tb927.3.2960, Tb927.3.3440, Tb927.4.2520, Tb927.4.2520, Tb927.4.4070, Tb927.5.1880, Tb927.5.300, Tb927.6.2390, Tb927.6.3600, Tb927.6.4320, Tb927.7.1110, Tb927.7.1200, Tb927.7.190, Tb927.7.190, Tb927.7.3770, Tb927.7.4570, Tb927.7.5160, Tb927.7.5540, Tb927.7.6270, Tb927.8.1910, Tb927.8.2210, Tb927.8.2540, Tb927.8.2610, Tb927.8.7380, Tb927.9.15070, Tb927.9.3280, Tb927.9.7550, Tb927.9.9000, TcCLB.408799.19, TcCLB.436521.9, TcCLB.447255.20, TcCLB.447255.20, TcCLB.503855.30, TcCLB.503899.90, TcCLB.503991.39, TcCLB.504137.70, TcCLB.504153.160, TcCLB.504171.40, TcCLB.504433.14, TcCLB.504703.10, TcCLB.506213.50, TcCLB.506363.70, TcCLB.506411.10, TcCLB.506411.10, TcCLB.506417.5, TcCLB.506457.60, TcCLB.506513.110, TcCLB.506605.170, TcCLB.506795.44, TcCLB.506811.190, TcCLB.506931.10, TcCLB.506941.120, TcCLB.506943.50, TcCLB.506943.80, TcCLB.507639.50, TcCLB.507689.30, TcCLB.507711.140, TcCLB.507757.70, TcCLB.507875.20, TcCLB.508169.30, TcCLB.508211.70, TcCLB.508533.40, TcCLB.508717.50, TcCLB.508771.10, TcCLB.508827.40, TcCLB.509151.130, TcCLB.509205.120, TcCLB.509647.180, TcCLB.509759.10, TcCLB.509829.20, TcCLB.509941.100, TcCLB.509965.330, TcCLB.510141.10, TcCLB.510187.380, TcCLB.510287.60, TcCLB.510293.70, TcCLB.510315.10, TcCLB.510507.20, TcCLB.510765.10, TcCLB.510785.20, TcCLB.510879.80, TcCLB.511127.160, TcCLB.511127.80, TcCLB.511577.110, TcCLB.511803.30

## Results

### Data mining and sequence retrieval

**Fig 1:**
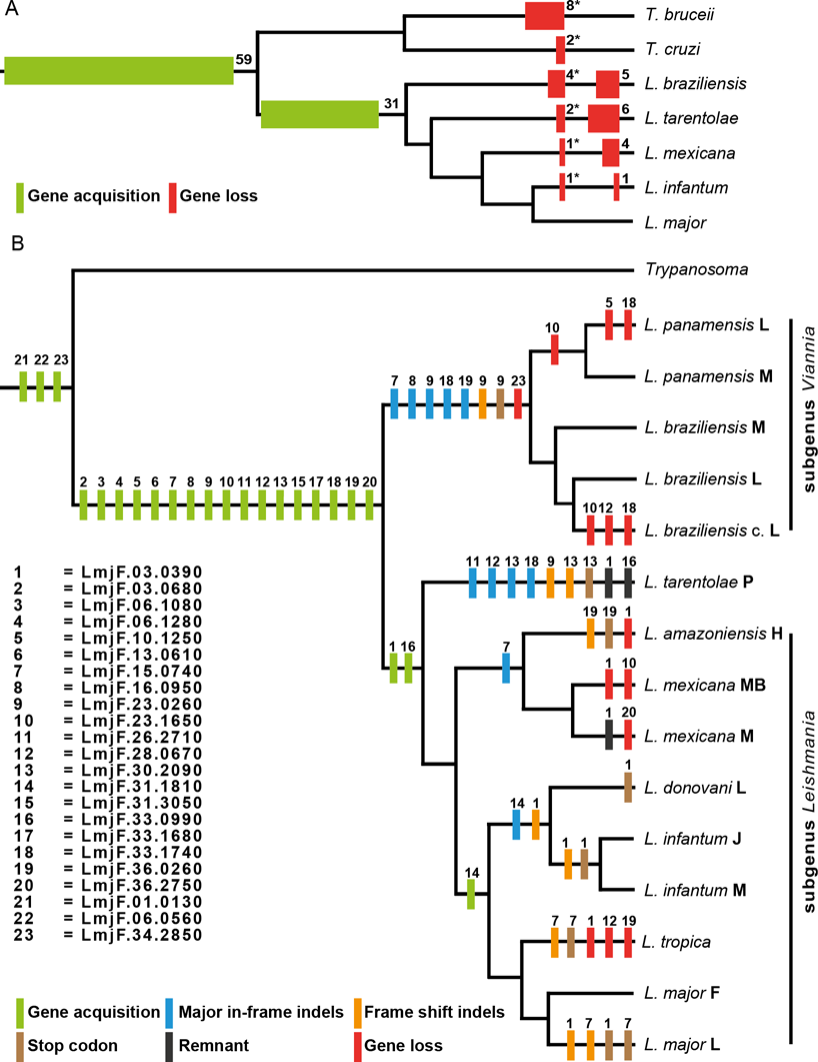
Consensus trees with orthologues to LGTs. **(A)** Phylogenetic tree (congruent with the systematics proposed by Fraga and co-workers from 2010) showing the presence of LGT orthologs in five sequenced genomes of *Leishmania* and the sequenced genomes of *Trypanosoma cruzi* and *Trypanosoma bruceii*. The number of acquired genes (green boxes) and lost genes (red boxes) are presented numerically. (Red boxes*) refers to gene loss of orthologs to universal trypanosomatid LGTs. **(B)** Consensus tree summarizing the agreement between the phylogenetic trees for the twenty-three LGTs, investigated in detail in this study. Genus *Leishmania* is divided into the three subgenera *Viannia*, *Sauroleishamania* (here represented by only one species and one strain–*L*. *tarentolae*) and *Leishmania*. Subgenera *Viannia* and *Leishmania* are further divided into several complexes, and the topology of the consensus tree is completely congruent with the systematics proposed by Fraga and co-workers (2010). The fate of each LGT is illustrated with coloured boxes. “Gene acquisition” (green boxes) and “Gene loss” (red boxes) are based on presence of orthologs in published genomes or amplification of orthologs with PCR. Insertions/deletions (indels) (blue and orange boxes) and presence of stop codons (brown boxes) have been determined by manual inspections of alignments of obtained sequences and are relative to the gene sequence in *L*. *major* F. “In-frame Indels” (blue boxes) are insertions or deletions of more than 50 nucleotides not causing any frame-shift. “Frame-shift Indels” (orange boxes) are insertions or deletions that causes frame-shifts. Remnants (black boxes) of orthologs have been identified with synteny analyses (see Material and Methods).

Fifty-nine of the LGTs previously confirmed in *L*. *major* [11, 12] were found to have orthologs also in close relatives, belonging to genus *Trypanosoma*, indicating that their transfer preceded the radiation of the trypanosomatids. We found that these universal trypanosomatid LGTs are, with a few exceptions, present in all published genomes of genus *Leishmania*–four are lost in *L*. *braziliensis*, two in *L*. *tarentolae*, and one each is lost in *L*. *mexicana* and *L*. *infantum*. In contrast, thirty-one LGTs detected in *L*. *major* only, and not found in any of the other trypanosomatids, exhibit a more heterogeneous distribution in genus *Leishmania*–five are lost in *L*. *braziliensis*, six in *L*. *tarentolae*, four in *L*. *mexicana* and one is missing from the genome of *L*. *infantum* (Fig 1A and S3 Table).

### Sequence similarity and G+C content

We have compared the nucleotide and amino acid identities, as well as the G+C contents, of the LGTs in *L*. *braziliensis*, *L*. *infantum* and *L*. *tarentolae* with corresponding values of the coding content of the complete genomes. The nucleotide and amino acid identities between the universal trypanosomatid LGTs ranges from 76.7% to 94.0% (nucleotide level), and from 76.6% to 93.5% (amino acid level), respectively. This resembles the nucleotide and amino acid identity scores between the published genomes of *L*. *major*, *L*. *braziliensis*, *L*. *infantum* and *L*. *tarentolae* ranging from 79.2% to 94.0% and from 74.8% to 92.0% respectively. In contrast, nucleotide and amino acid identities in orthologs to the LGTs unique to genus *Leishmania* are significantly lower (p-value <0.05) than those of the genomes, ranging from 68.7% to 92.1% and from 66.8% to 88.8% respectively (Table 2).

**Table 2:** Nucleotide and amino acid identity comparisons.

Species compared	Nucleotide identity			Amino acid identity		
	Genome	Universal trypanosomatid LGTs	Genus *Leishmania* unique LGTs	Genome	Universal trypanosomatid LGTs	Genus *Leishmania* unique LGTs
***L*. *major—L*. *infantum***	94.0%^a^	94.0%	92.1%	92.0%^a^	93.5%	88.8%
***L*. *major—L*. *tarentolae***	84.9%^b^	82.9%	77.8%	81.9%^b^	82.5%	77.8%
***L*. *major—L*. *braziliensis***	82.0%^a^	82.2%	77,6%	77.0%^a^	80.4%	74.5%
***L*. *infantum—L*. *tarentolae***	85.0%	84.4%	78.0%	82.4%^b^	84.4%	78.0%
***L*. *infantum—L*. *braziliensis***	81.0%^a^	82.2%	78.0%	77.0%^a^	80.5%	74.9%
***L*. *tarentolae—L*. *braziliensis***	79.2%^b^	76.7%	68.7%	74.8%^b^	76.6%	66.8%

^a^ [28]

^b^ [29]

The average G+C-content of orthologous universal trypanosomatid LGTs, as well as the average G+C-contents of the LGTs unique to genus *Leishmania*, is lower than the coding G+C-content of the *L*. *major*, *L*. *braziliensis*, *L*. *infantum* and *L*. *tarentolae* complete genomes. However, the differences are at the most only 1.6% (*L*. *major*: 62.5%-60.9%) and 3.4% (*L*. *infantum*: 62.5%-59.1% and *L*. *major* 62.5%-59.1%) respectively, and not statistically significant (p<0.05) (Table 3).

**Table 3:** G+C content of coding content of complete genomes and in sets of LGTs.

	G+C content in coding sequence
***L*. *braziliensis* (genome)**	60.4%^a^
**Universal trypanosomatid LGTs in *L*. *braziliensis***	59.1%
**Genus *Leishmania* unique LGTs in *L*. *braziliensis***	57.2%
***L*. *infantum* (genome)**	62.5%^a^
**Universal trypanosomatid LGTs in *L*. *infantum***	61.0%
**Genus *Leishmania* unique LGTs in *L*. *infantum***	59.1%
***L*. *major* (genome)**	62.5%^a^
**Universal trypanosomatid LGTs in *L*. *major***	60.9%
**Genus *Leishmania* unique LGTs in *L*. *major***	59.1%
***L*. *tarentolae* (genome)**	58.4%^a^
**Universal trypanosomatid LGTs in *L*. *tarentolae***	58.3%
**Genus *Leishmania* unique LGTs *L*. *tarentolae***	56.5%

^a^ [29]

### Distribution of LGTs in genus *Leishmania*

To deepen our study of LGT in genus *Leishmania* we used a PCR based approach to determine the presence of orthologs to twenty of the LGTs initially detected in *Leishmania major* but missing from the genomes of *Trypanosoma brucei* and *Trypanosoma cruzi*. We investigated ten additional strains of *Leishmania*, all with unpublished genomes (Table 1), and three universal trypanosomatid LGTs were added for comparison (S4 Table). Since the divergence of completely sequenced genomes defines the outer borders of our dataset, primers for PCR to detect the LGTs in species not yet sequenced, can be designed within conserved regions, and are thus likely to amplify orthologous regions within our dataset. The presence of orthologs in different strains is defined by sequence homology, with a minimum of 90% nucleotide identity to a sequence in a published *Leishmania* genome. This is well above the so called twilight zone for identification based on sequence homology [46]. We found that eleven of the twenty LGTs have orthologs in all fifteen strains investigated in this study, and that further six LGTS have orthologs in all three subgenera although one or several strains may be affected by loss of LGTs. Three LGTs; LmjF03.0390, LmjF31.1810 and LmjF.33.0990, only have orthologs in subgenus *Leishmania*, and of these LmjF.31.1810 only have orthologs in the *donovani-*, *major*- and *tropica*-complexes. Two out of the three universal trypanosomatid LGTs, LmjF.01.0310 and LmjF.06.0560, have orthologs in all fifteen strains included in this study, while the remaining ortholog, LmjF.34.2850, lack orthologs in subgenus *Viannia* (S4 Table).

Phylogenetic analysis of available data for each separate gene was performed, and all most parsimonious trees obtained from the separate analyses were used to construct a reference tree, shown in Fig 1B. The reference tree is completely congruent with the systematics proposed by Fraga and co-workers (2010). We also identified remnants of orthologs to the LGTs in already published genomes by analysing intergenic regions between syntenic orthologs of the flanking genes of the LGTs in *L*. *major*. Our aim was to be able to date the uptake of these LGTs to before, or after, the divergence of genus *Leishmania* into different subgenera. Based on presence in different strains of *Leishmania* (S4 Table), seventeen of the twenty LGTs appear to have been transferred after the divergence of genus *Leishmania* from other trypanosomatids, but before the division into the three subgenera present to day (Fig 1B). Three LGTs; LmjF03.0390, LmjF31.1810 and LmjF.33.0990, appears to have been transferred after the division of the *Leishmania* genus into the three subgenera, since LmjF.03.0390 and LmjF.33.0990 lack orthologs in subgenus *Viannia*, and LmjF.31.1810 only have orthologs in the *donovani*, *major* and *tropica* complexes (Fig 1B and S4 Table). However, there are remnants of orthologs to LmjF.03.0390 and LmjF.33.0990 in *L*. *tarentolae* strain P, with nucleotide identities of 34% and 63% respectively, suggesting that these two LGTs have been transferred after the divergence of subgenera *Leishmania* and *Sauroleishmania* from subgenus *Viannia*. A remnant of LmjF.03.0390 was also detected in *L*. *mexicana* strain M (60% identity on the nucleotide level). In contrast, although the genes flanking LmjF.31.1810 have syntenic orthologs in the published genome of *L*. *mexicana*, there are no obvious signs of any remnants of an ortholog to LmjF.31.1810 in *L*. *mexicana*. The transfer of LmjF.31.1810 is thus inferred to have occurred after the divergence of the *mexicana* complex from the other complexes in subgenus *Leishmania* (Fig 1B).

We have also analysed the orthologs of the LGTs with respect to presence of stop codons, frame shift nucleotide insertion or deletions (indels) and major (comprising more than fifty nucleotides) nucleotide indels. Ten of the twenty LGTs are intact and of similar length in all orthologs investigated. Five LGTs have orthologues affected by major in-frame indels, while five LGTs are also affected by internal stop codons and/or major framshift indels (Fig 1B).

### Rate of sequence substitution

**Fig 2:**
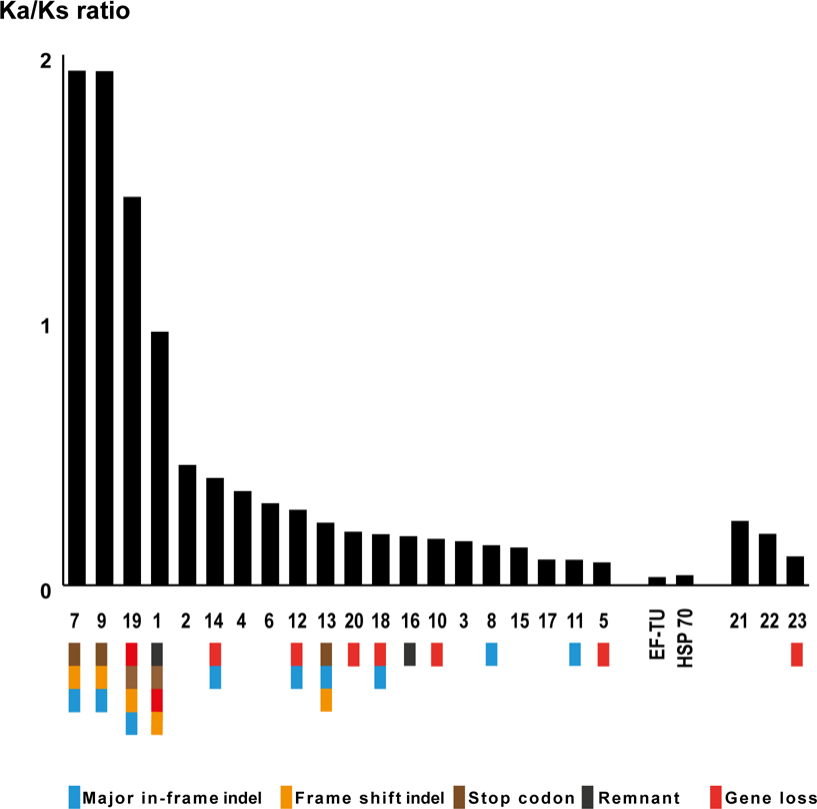
Average K_a_/K_s_ ratios between the LGTs. The average K_a_/K_s_ ratios for each of the LGTs and reference genes between strains. The numbers under the bars in represent the LGTs as listed in Fig 1A. The boxes under the bars shows the fate of the corresponding LGT in genus *Leishmania*–classified as gene acquisition, degradation, acquirement of stop codons and indels respectively. Four LGTs (LmjF.03.0390, LmjF.15.0740, LmjF.23.0260 and LmjF.36.0260) have K_a_/K_s_ ratios ≥1, and these LGTs have frame shift- and major in-frame indels, and contain stop codons.

In order to determine if the acquired LGTs are under neutral, positive, or negative selection in the different strains of *Leishmania*, the evolutionary rate of LGTs was estimated by calculating the non-synonymous over synonymous substitutions (K_a_/K_s_ ratio) for pairwise alignments of all orthologs of LGTs. To put the rates into perspective we also calculated the K_a_/K_s_ ratio for a set of reference genes (S2 Table). As such, K_a_/K_s_ ratios of reference genes were all determined to be less than one. In three LGTs; LmjF.15.0740, LmjF.23.0260 and LmjF.36.0260, the K_a_/K_s_ ratios are greater than one, and these three LGTs are also interrupted by stop codons in one or more species. In addition, one LGT, LmjF.03.0390 detected only in subgenus *Leishmania*, display K_a_/K_s_ ratios close to one, and all detected orthologs to LmjF.03.0390 are degraded to some extent (Fig 2). In the remaining LGTs, K_a_/K_s_ ratios are less than one but greater than the K_a_/K_s_ ratios of the reference genes. Detailed information can be found in S5 Table.

### Codon bias calculation

To elucidate whether the LGTs share patterns similar to those of genes determined to be functional in *Leishmania* [43, 44, 47] the codon bias was determined using Codon Adaptation Index (CAI) scores [48]. The mean CAI scores for the majority of orthologous LGTs show no significant variations indicating that these genes are expressed. However, three cases; LmjF.15.0740, LmjF.23.0260 and LmjF.36.0260, display extensive codon bias variations (high coefficient of variance), indicated in bold text in Table 4. A closer inspection reveals that these three LGTs have a subset of orthologs with significantly lower average CAI-scores than the remaining orthologs (pooled two-sample t-test, p<0,001). Additionally, LmjL.15.0740 and LtrX.15.0740, orthologs to LmjF.15.0740, and all orthologs to LmjF.23.0260 in subgenus *Viannia* contain stop codons. None of the orthologs to LmjF.36.0260 in subgenus *Viannia*, are interrupted by stop codons, however these orthologs are distinctly diverged from the orthologs in subgenera *Leishmania* and *Sauroleishmania*, in respect to nucleotide sequence similarity (sequence identity for orthologs within subgenus *Viannia* >97%, sequence identity between orthologs in subgenus *Viannia* and remaining orthologs <85%). The *Leishmania amazoniensis* strain H ortholog, LamH.36.0260, contains several stop codons and, consequently, the CAI score is the lowest among the scores of orthologs belonging to subgenus *Leishmania*.

**Table 4:** CAI scores of LGTs.

LGT	Average CAI	CV (coefficient of variance)
**LmjF.01.0310** ^b^	0.58	5.31%
**LmjF.03.0390**	0.57	6.87%
**LmjF.03.0680**	0.66	4.13%
**LmjF.06.1080**	0.71	5.25%
**LmjF.06.1280**	0.52	4.24%
**LmjF.06.0560** ^b^	0.63	2.94%
**LmjF.10.1250**	0.64	2.66%
**LmjF.13.0610**	0.69	5.38%
**LmjF.15.0740**^a^	0.67	9.53%
**LmjF.16.0950**	0,59	3.32%
**LmjF.23.0260**^a^	0.70	15.68%
**LmjF.23.1650**	0.68	5.43%
**LmjF.26.2710**	0.66	4.08%
**LmjF.28.0670**	0.63	2.94%
**LmjF.30.2090**	0.69	3.94%
**LmjF.31.1810**	0.76	1.16%
**LmjF.31.3050**	0.65	1.20%
**LmjF.33.0990**	0.73	2.16%
**LmjF.33.1680**	0.67	3.45%
**LmjF.33.1740**	0.62	4.40%
**LmjF.34.2850**^b^	0.66	2.43%
**LmjF.36.0260**^a^	0.67	14.47%
**LmjF.36.2750**	0.64	3.42%

^a^ Average CAI scores with large variations

^b^ Universal trypanosomatid LGTs

**Fig 3:**
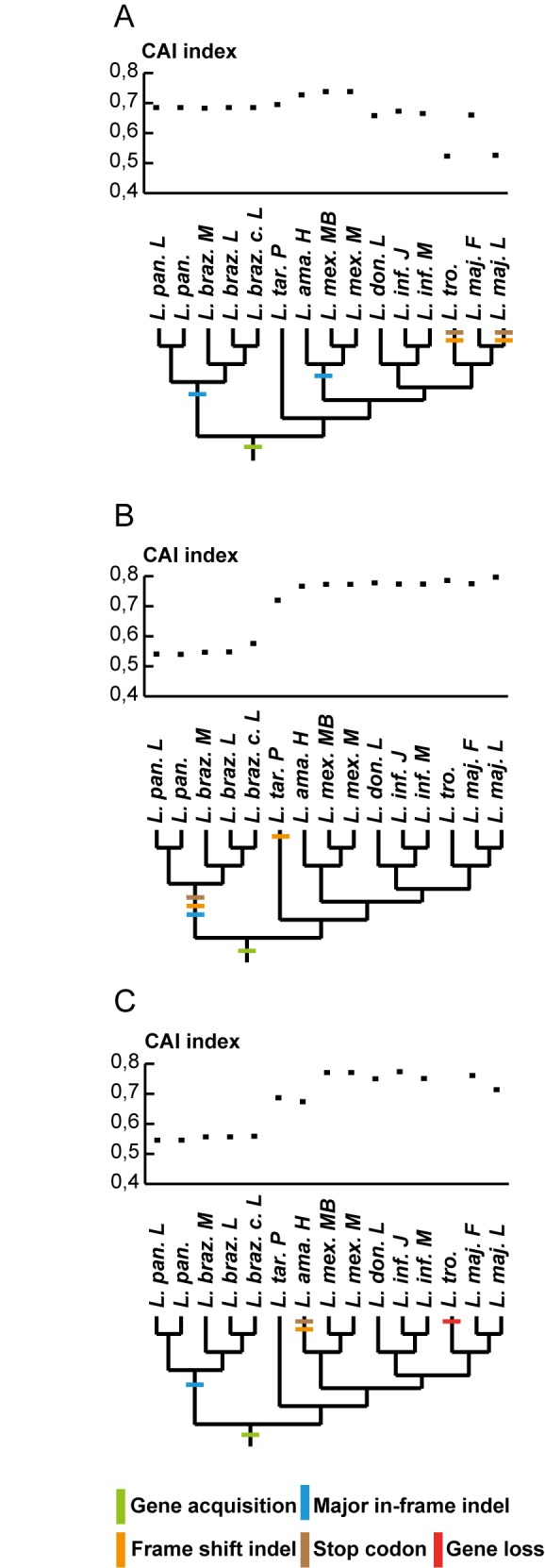
LGTs with large variations in codon bias among their orthologs. (A) CAI scores of orthologues to LmjF.15.0740, and the fate of the LGT in genus *Leishmania*. The average CAI score of LmjL.15.0740 and LtrX.15.0740 is significantly lower than the average CAI score of the other orthologs to LmjF.15.0740 (p<0,001). Frame shift indels and stop codons in LmjL.15.0740 and LtrX.15.0740, as well as major in-frame indels in subgenus *Viannia* and *mexicana-*complex are indicated in the phylogenetic tree. (B) CAI scores of orthologues to LmjF.23.0260, and the fate of the LGT in genus *Leishmania*. The average CAI scores of orthologs to LmjF.23.0260 in subgenus *Viannia* is significantly lower than the average CAI scores of the other orthologs to LmjF.23.0260 (p<0,001). Major in-frame indels, frame shift indels and stop codons in all orthologs of subgenus *Viannia*, as well as stop codon in *L*. *tarentolae* are indicated in the phylogenetic tree. (C) CAI scores of orthologues to LmjF.36.0260, and the fate of the LGT in genus *Leishmania*. The average CAI scores of orthologs to LmjF.36.0260 in subgenus *Viannia* is significantly lower than the average CAI scores of the other orthologs to LmjF.23.0260 (p<0,001). LamH.36.0260 displays the lowest CAI score among orthologs of subgenus *Leishmania*. Major in-frame indels contributing to divergence of orthologs of subgenus *Viannia*, frame shift indels and stop codons in LamH.36.0260 and gene loss in *L*. *tropica* are indicated in the phylogenetic tree. Significance in panel A-C was calculated with a pooled two-sample t-test.

The CAI score for individual orthologs of LmjF.15.0740, LmjF.23.0260 and LmjF.36.0260, together with phylogenetic trees showing the fate of the LGT in genus *Leishmania* for each of the LGTs, are presented in Fig 3.

## Discussion

In this study we contribute data to increase the understanding of the fate of genes acquired by LGT in eukaryote genomes. We have combined a PCR based screen and sequence determination of orthologues to previously detected LGTs in un-sequenced *Leishmania* genomes with thorough bioinformatics analyses of our unique dataset. Our aim was to determine the depth of the transfer of LGTs previously reported in *L*. *major* within genus *Leishmania* and to evaluate the fate of the *Leishmania*-specific transferome in comparison to the LGTs shared by all trypanosomatids.

The *L*. *major* genome contains about 8300 genes [10] and approximately 6200 of these are shared with the closely related trypanosomatids; *T*. *brucei* and *T*. *cruzi*. Less than 1000 genes are unique to *L*. *major* [12]. The number of universal trypanosomatid LGTs has been estimated to fifty-nine while thirty-one LGTs appeared to be unique to genus *Leishmania* (S3 Table). Hence less than 1% of the universal trypanosomatid genes are of prokaryote origin and these are, with a few exceptions, present in all published *Leishmania* genomes. However, 3% of the genes unique to *L*. *major* are LGTs. The LGTs uniquely detected in genus *Leishmania*, display a more heterogeneous distribution, with a relatively high degree of gene loss, compared to the shared trypanosomatid set of LGTs. Comparisons of the published *Leishmania* genomes reveal that the number of species specific genes are surprisingly low, considering the early diversification of genus *Leishmania* [28–30]. Our results hence indicate that LGTs contributes to speciation as well as species diversification within genus *Leishmania*.

Comparative studies of the published genomes of *Leishmania* also unambiguously show that they are similar with respect to G+C-content, nucleotide composition and amino acid content [28–30]. Our analyses show that the G+C content of universal trypanosomatid LGTs and the LGTs uniquely present in genus *Leishmania* resemble that of the recipient genomes. Anomalous G+C content of genes have been used as tools to identify LGTs [49, 50]. However, previous surveys have concluded that comparisons of G+C-content alone are poor markers for LGT, due to the effects of amelioration [51, 52]–the process by which foreign DNA adapts to resemble the nucleotide composition of the host genomes [49]. Our results indicate that LGTs detected in genus *Leishmania* have ameliorated so that the G+C content resemble that of the recipient genomes. Notably, comparisons of nucleotide and amino acid identities between the orthologs to LGTs detected in published *Leishmania* genomes reveal that the universal trypanosomatid LGTs are more conserved than the LGTs present in genus *Leishmania* only. A likely consequence of have resided in their host genomes a longer time period than the species specific LGTs. Consequently the LGTs detected in *L*. *major*, but not found in any other trypanosomatids, were most likely transferred after the divergence of genus *Leishmania*, rather than before speciation of the trypanosomatides and then subsequently lost in the trypanosomes. These observations also support the indication that LGTs contribute to speciation and species diversification in genus *Leishmania*.

To further determine the depth and dynamics of LGTs in genus *Leishmania*, we have explored and analysed the gene sequences of orthologs to twenty LGTs unique to genus *Leishmania* in ten additional strains from eight species of *Leishmania*. Three universal trypanosomatid LGTs were also included, for comparison. Presence of LGTs in each genome as well as stop codons and intragenic indels were mapped onto a phylogenetic tree (Fig 1B), showing that seventeen of the LGTs were acquired prior to the division of the *Leishmania* genus. Eleven of these have intact orthologs in all strains investigated, and are thus assumed to be fixed in each respective genome. Two genes appear to have been acquired after the divergence of subgenus *Viannia* from subgenera *Leishmania* and *Sauroleishmania*, and one single gene even later than that–after the divergence of the *mexicana* complex from the *donovani*, *tropica* and *major* complexes. Two out of the three universal trypanosomatid LGTs are distributed throughout the entire genus *Leishmania*, while one is absent in subgenus *Viannia* (Fig 1B). Altogether our results indicate that LGT in genus *Leishmania* is an ongoing process, and they also support the conclusion that LGT contributes to successive species-specific diversifications.

LGTs contribute to genome evolution in prokaryotes [14, 15] and eukaryotes alike [16–18]. Recent studies have shown that the fate of LGTs in eukaryote genomes are dependent on the new capacities that they are providing to the organism–beneficial genes are conserved and superfluous genes are discarded [24].

To evaluate the rate of evolution we have determined whether the LGTs are under neutral, positive or negative selection in the genomes of the different strains of *Leishmania* by comparison of synonymous and non-synonymous substitutions in orthologous LGTs. We also analysed a set of so called housekeeping genes, chosen as references. K_a_/K_s_ ratios between orthologs of reference genes confirm that they are under purifying selection. In a similar fashion, sixteen *Leishmania* specific LGTs, and all three of the universal trypanosomatid LGTs, display K_a_/K_s_ ratios indicative of conservation. However, the remaining four LGTs evolve under a low selective pressure (Fig 2). Among these four genes we detected orthologs interrupted by stop codons, a direct indication of relaxed pressure. The variation in conservation among LGTs strengthens the conclusion that LGT in genus *Leishmania* is indeed a dynamic process, facilitating diversification.

Comparative studies of gene expression in *Leishmania* indicate that the majority of genes are constitutively expressed by means of polycistronic transcription [43, 53, 54]. Differentiation of expression is thus hypothesized to be post-transcriptional, and partly facilitated via codon usage bias [44]. Synonymous codon usage bias can be estimated by calculation of CAI scores [48], which has been used to predict expression levels of all genes in the close relative *Trypanosoma cruzi* [44]. These predictions were confirmed by comparisons to the complete proteome of *T*. *cruzi* [55], and it has also been concluded that relative codon bias is conserved among the trypanosomatids [45]. We have calculated the CAI scores of orthologous LGTs, with the purpose to evaluate whether LGTs may be expressed by the parasite or not. The mean CAI scores for the majority of LGTs show no significant variations and fall in the range of the expression levels of genes in tandem–*i*.*e*. highly expressed genes [10, 30]. However, three LGTs (LmjF.15.0740, LmjF.23.0260 and LmjF.36.0260) display large variations in average CAI scores (Table 4). Our further comparisons show that LGTs displaying large average CAI score variations evolve with high evolutionary rates and show signs of either degradation or gene innovation (Fig 2). In conclusion, the dynamics of LGT in genus *Leishmania* correlates with predicted expression levels.

The impact of LGTs on prokaryotes has been extensively studied [14, 15], and it is now clear that LGTs from prokaryotes have an influence on metabolic pathways in eukaryotic microorganisms, either by homologue replacement or by providing new capacities to the organism [11, 16–18]. It is also clear that gene transfer that are not beneficial for the recipient genomes will be either lost or modified *i*.*e*. even though the uptake of LGTs is assumed to be a random process, the fixation of foreign genes depend on selective evolutionary pressure. [21, 24, 56]. It has also been suggested that LGTs is a paramount mechanism for gene innovation [57]. Our data set contains examples of conserved LGTs as well as LGTs that are evolving fast. Among fast evolving LGTs we have observed genes potentially developing new functions, but also genes that are being degraded. Among our data set of 20 LGTs, eleven LGTs have been assigned a predicted function (S4 Table). However, the remaining nine LGTs encode hypothetical proteins, and eight of these are both conserved and predicted as highly expressed. This highlights the importance of further investigations on neglected pathogens to elucidate the function of uncharacterized proteins and their impact on organism adaptation and diversity. In conclusion, when a gene is acquired there are three possible scenarios; conservation, degradation or innovation. If orthologues of the same ancestral gene takes on different routes of evolution, these differences in the metabolic repertoire may fuel speciation. In our dataset we detected genes that show features indicative of all these three different scenarios.

For example, LGT LmjF.16.0950 encodes a sucrose-phosphate synthase-like protein, which converts sucrose to fructose–a primary energy sources for promastigotes residing in the sandfly [58]. The sucrose-phosphate synthase-like protein is, according to our survey, widely distributed in the genus *Leishmania* and conserved as well as predicted as highly expressed. Hence a result of a gene transfer facilitating adaptation to the life stages in the insect vector and thus generally beneficial for the parasites. Macrophages have many functions in the human body, one being the removal of erythrocytes from the blood. This causes a release of heme into the phagolysosome, where it is degraded and proposed to be utilized by *Leishmania* amastigotes in synthesis of heme-proteins. All trypanosomatides are auxotrophs for heme, but *Leishmania* spp. only harbours genes encoding the last three steps of the hemeproduction. The first two steps are catalysed by coproporphyrinogen-III oxidase followed by protoporphyrinogen oxidase (PPOX), both proteins encoded by LGTs [58]. PPOX (LmjF.06.1280 and orthologs) is distributed and conserved in the entire genus *Leishmania*, however, the average CAI scores are the lowest (0.52) among the average CAI scores of all the studied LGTs. This predicted, limited expression of LmjF.06.1280 correlates with the function of the gene, since it is suggested to be important only during a short period in the life cycle of *Leishmania* parasite.

Another example is LmjF.36.0260 that encodes xyuolokinase, an enzyme that together with ribulokinase and ribokinase is involved in the metabolism of C5-sugars. This acquisition allows *Leishmania* parasites to assimilate nutrients from other types of sugars than the ones present in the diet of the sand fly [58] and is thus important for the adaptation to the life stages in the human host. The divergence between the LmjF.36.0260 orthologs in subgenus *Viannia* and the orthologs in the other subgenera together with the K_a_/K_s_ ratios for these orthologs, (Fig 2) indicate that either these LGTs are being degraded, or the groups are diverging from each other with higher rates than expected. The latter of these two alternatives is supported by the fact that none of the orthologs except LamH.36.0260 contain stop codons. The CAI scores of the orthologs in subgenus *Leishmania* and subgenus *Sauroleishmania* indicates that these genes are highly expressed, hence the possible degradation of LamH.36.0260 may be in an early stage. The comparably low CAI scores for orthologs in subgenus *Viannia* (Fig 3C) suggest that the function of LmjF.36.0260 in the three different subgenera may not be the same. It seems as if this additional enzyme for metabolizing C5-sugars is still beneficial for the majority of the strains in subgenus *Leishmania* and in subgenus *Sauroleishmania*, but maybe less so in the strains of subgenus *Viannia*. Thus, this may be an example of an LGT going down a route for gene innovation. In addition, LmjF.26.2710 encodes a glutamate-5-kinase, which is involved in the metabolism of amino groups. The relaxed constraints of evolution inferred in orthologs of subgenus *Viannia* might be indicative of diversifying selection possibly leading to novel protein functions among orthologues in subgenus *Viannia*.

LmjF.23.0260 encodes argininosuccinate synthetase, which is active in the urea cycle. *Leishmania* parasites secretes urea in the phagolysosome to sustain the low pH that is required for the differentiation of promastigotes into amastigotes [59–61]. The LmjF.23.0260 orthologs in subgenera *Leishmania* and *Sauroleishmania* lack stop codons, however the high K_a_/K_s_ ratios (Fig 2) and predicted high expression levels (Fig 3B), indicate that these orthologs are possibly diverging to acquire new functions. The orthologs to LmjF.23.0260 in strains from subgenus *Viannia* harbour stop codons and exhibits K_a_/K_s_ ratios >1 (Fig 2). The predicted levels of expression are also lower than those of other LmjF.23.0260 orthologs (Fig 3B), indicating that all of the subgenus *Viannia* orthologs may be degrading. In addition, LmjF.15.0740 (a hypothetical protein) shows signs of degradation by means of stop codons and low CAI values in some species.

LGTs have previously been suggested as potential drug targets [62] and our study highlights the importance of paying attention to the dynamics of LGTs while selecting such genes as targets for new drugs to hamper infection. In conclusion, our results show that universal trypanosomatid LGTs are widely distributed and conserved in genus *Leishmania*, which is a likely consequence of the long time period that the universal trypanosomatid LGTs have resided in their respective recipient genomes. The LGTs that are uniquely present in genus *Leishmania* are more divergent than the rest of the genomes, which either indicates that many of these LGTs and their orthologs have not yet ameliorated to resemble the host genomes, or that they are evolving at a faster rate than the recipient genomes. The latter scenario is supported by our observations of high variations in nucleotide and amino acid composition, high rates of evolution and pronounced codon bias. In addition several examples of differential pseudogenization among these LGTs can be inferred; *i*.*e*. genes are acquired, initially found beneficial for one specific lineage (and thus fixated in closely related strains), but later rendered superfluous, or modified in some strains, yet conserved in others. LGT in genus *Leishmania* is thus an ongoing continuous and dynamic process where gains and losses of genes occur as the lineage evolves and diverges. We thus suggest that LGTs contribute to the shaping and speciation of genus *Leishmania*.

## Supporting Information

S1 Table Primers used. Information about primers used to amplify the orthologs to the LGTs investigated in this study. The information includes primer sequences, annealing temperatures and expected size for primer pairs.
(XLSX)

S2 Table Reference genes. Information about the reference genes used for comparisons of K_a_/K_s_ ratios.
(XLSX)

S3 Table Presence of LGTs in different strains of *Leishmania*. A compilation of orthologs to LGTs previously confirmed with phylogenomic analyses in *L*. *major*. Fifty-nine have orthologs also in close relatives belonging to genus *Trypanosoma*, and thirty-one have only orthologs in genus *Leishmania*.
(XLSX)

S4 Table Presence of LGTs in different strains of *Leishmania*. A compilation of the twenty-three LGTs deeply investigated in this study, and the presence of orthologs to these genes in the fifteen strains included in this study.
(XLSX)

S5 Table Results from K_a_/K_s_ calculations. Results from calculations for pairwise alignments of all orthologs obtained for each of the studied LGTs, using the modified Yang-Nielson method (MYN) [40] incorporated in the software package Ka/Ks_Calculator [41].
(XLSX)
